# Cell Intrinsic IL-38 Affects B Cell Differentiation and Antibody Production

**DOI:** 10.3390/ijms24065676

**Published:** 2023-03-16

**Authors:** Arnaud Huard, Christian Wilmes, Anastasiia Kiprina, Christoph Netzer, Gaby Palmer, Bernhard Brüne, Andreas Weigert

**Affiliations:** 1 Institute of Biochemistry I, Faculty of Medicine, Goethe-University Frankfurt, 60590 Frankfurt, Germany; 2 Division of Rheumatology, Department of Medicine, Faculty of Medicine, University of Geneva, 1211 Geneva, Switzerland; 3 Department of Pathology and Immunology, Faculty of Medicine, University of Geneva, 1211 Geneva, Switzerland; 4 Department of Otorhinolaryngology, Head and Neck Surgery, University Medical Center Göttingen, 37075 Göttingen, Germany; 5 Geneva Center for Inflammation Research, Faculty of Medicine, University of Geneva, 1211 Geneva, Switzerland; 6 Fraunhofer Institute for Translational Medicine and Pharmacology ITMP, 60596 Frankfurt, Germany; 7 Frankfurt Cancer Institute, Goethe-University Frankfurt, 60596 Frankfurt, Germany; 8 German Cancer Consortium (DKTK), Partner Site Frankfurt, 60590 Frankfurt, Germany; 9 Cardio-Pulmonary Institute (CPI), 60590 Frankfurt, Germany

**Keywords:** IL-1 family, IL-38, B cell differentiation, autoimmunity, antibodies

## Abstract

IL-38 is an IL-1 family receptor antagonist with an emerging role in chronic inflammatory diseases. IL-38 expression has been mainly observed not only in epithelia, but also in cells of the immune system, including macrophages and B cells. Given the association of both IL-38 and B cells with chronic inflammation, we explored if IL-38 affects B cell biology. IL-38-deficient mice showed higher amounts of plasma cells (PC) in lymphoid organs but, conversely, lower levels of plasmatic antibody titers. Exploring underlying mechanisms in human B cells revealed that exogenously added IL-38 did not significantly affect early B cell activation or differentiation into plasma cells, even though IL-38 suppressed upregulation of CD38. Instead, IL-38 mRNA expression was transiently upregulated during the differentiation of human B cells to plasma cells in vitro, and knocking down IL-38 during early B cell differentiation increased plasma cell generation, while reducing antibody production, thus reproducing the murine phenotype. Although this endogenous role of IL-38 in B cell differentiation and antibody production did not align with an immunosuppressive function, autoantibody production induced in mice by repeated IL-18 injections was enhanced in an IL-38-deficient background. Taken together, our data suggest that cell-intrinsic IL-38 promotes antibody production at baseline but suppresses the production of autoantibodies in an inflammatory context, which may partially explain its protective role during chronic inflammation.

## 1. Introduction

Interleukin-38 (IL-38) is a member of the IL-1 cytokine family, belonging to the IL-36 subfamily and sharing homology with the IL-1 family receptor antagonists (Ra)—IL1Ra and IL-36Ra [1]. As for the other IL-1 family proteins, IL-38 plays a role in immunological processes, particularly in the control of inflammation, where it appears to act predominantly as a receptor antagonist. However, IL-38 has also been associated with non-immunological processes, such as the control of keratinocyte differentiation. To that end, IL-38 may not only act through its putative receptors, since high levels of IL-38 are found in the cytoplasm of keratinocytes [2,3,4]. Concerning inflammatory signaling, an initial study in 2012 proposed IL-38 as an anti-inflammatory mediator, based on its capacity to dampen Th17 responses triggered by *C. albicans*. Indeed, IL-38 is shown to regulate IL-17 production with similar kinetics when compared to IL-36Ra and shows opposite effects to IL-36, thus indicating that IL-38 may antagonize the IL-36 receptor (IL-36R) [5]. Another putative IL-1 family receptor for IL-38 was suggested recently. Hereby, IL-38 is released from apoptotic cells to limit macrophage activation and downstream T cell IL-17 production by blocking X-linked IL-1 receptor accessory protein-like 1 (IL-1RAPL1) signaling [2,6]. In addition to these two receptors, the IL-1 receptor (IL-1R) is also discussed to recognize IL-38 [7]. Thus, the predominant IL-38 receptor remains to be determined.

IL-38 is constitutively expressed in the skin, predominantly in the epidermis, as well as in some lymphoid organs, where it is found particularly in B cells and macrophages [1,2,3,8,9]. In fact, the first study describing IL-38 found IL-38 expression in proliferating B cells in tonsils [1]. Moreover, IL-38 plasma concentrations correlate positively with circulating memory B cells and plasmablasts, as well as stimulation of peripheral B cells, but not B cell-depleted PBMCs with rituximab-triggered IL-38 secretion [10]. Moreover, IL-38 in plasma, presumably derived from B cells, correlates negatively with the body mass index and metabolic syndromes in humans [10]. These studies clearly indicated that B cells produce IL-38. There are further suggestions of a functional role of IL-38 in B cells, particularly in the context of inflammatory bowel diseases (IBD) [11,12]. In colonic biopsy samples of healthy individuals and patients with IBD, including ulcerative colitis and Crohn’s disease, B cells were identified by immunostaining as the major cellular source of IL-38 in the colonic mucosa, while other immune cells, such as T cells or macrophages, did not produce IL-38 [11,12]. In this context, IL-38 was shown to reduce intestinal inflammation during DSS-induced colitis, by inhibiting the release of inflammatory mediators, supposedly from macrophages [11]. In addition, we previously showed that IL-38 in mice may affect B cell homeostasis during experimental autoimmune encephalomyelitis (EAE). IL-38 KO mice showed lower levels of B cells and plasma cells in the periphery while having higher levels of B cells in the spleen [9]. Thus, B cells may not only produce but also respond to IL-38.

B cells are main effector cells of the adaptive immune system. They provide a unique, high-affinity immune response and ensure long-term immunity against pathogens. The molecular processes of B cell activation are, therefore, strictly regulated and complex. Consequently, disturbances in these regulatory mechanisms are causatively involved in a variety of diseases, including malignancies and autoimmunity. In autoimmune diseases, B cells play a crucial role not only by producing autoantibodies, but also by presenting autoantigens, secreting inflammatory cytokines and contributing to other pathological mechanisms [13,14,15,16].

IL-38 polymorphisms are associated with increased susceptibility for autoimmune diseases such as psoriasis, spondyloarthritis, rheumatoid arthritis (RA), psoriatic arthritis, and systemic-lupus-erythematosus (SLE) [17,18,19,20]. B cells are involved in different ways in most of these diseases. Indeed, their role in SLE appears to be mostly related to their production of anti-DNA autoantibodies [21,22]. In IBD, B cells produce anti-granulocyte-macrophage colony-stimulating factors or anti-tropomyosin autoantibodies [23,24], while in RA, their functional relevance is related to their ability to present antigens for T cell activation [25,26,27]. However, the role of IL-38 in B cell biology in such disease settings or in general was not explored so far. We therefore set out, as the first step, to investigate the roles of exogenous and endogenous IL-38 in B cell activation and differentiation into antibody-producing plasma cells.

## 2. Results

### 2.1. Impaired Plasma Cells Homeostasis in IL-38-Deficient Mice

**Figure 1:**
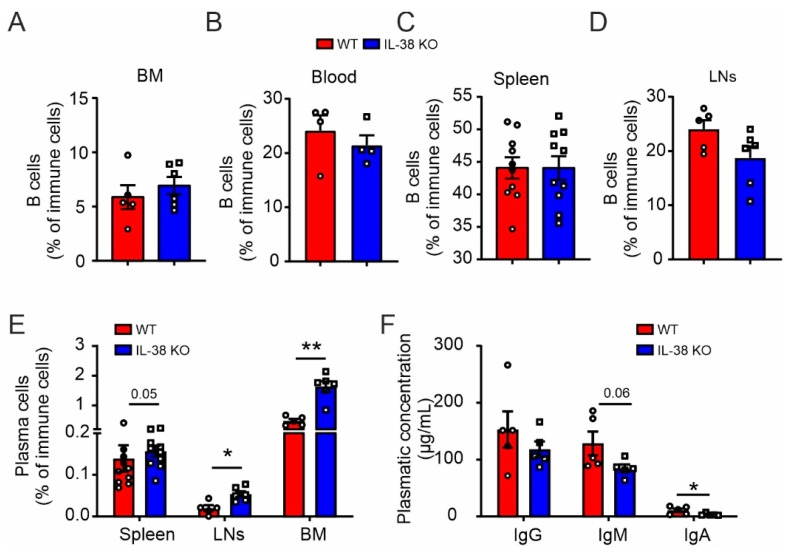
Baseline B cell numbers and antibody production in IL-38 KO mice. Flow cytometry was used to measure total B cell levels in bone marrow (BM) (**A**), blood (**B**), spleen (**C**), and lymph nodes (LNs) (**D**), and plasma cell levels (**E**) in different organs of WT and IL-38 KO mice. ELISA was used to measure plasmatic levels of IgG, IgM, and IgA in WT and IL-38 KO mice (**F**). Each data point corresponds to one animal. Data are means ± SEM. * *p* < 0.05; ** *p* < 0.01. *p*-values were calculated using multiple *t*-tests with FDR correction (**E**,**F**).

We first analyzed the impact of IL-38 on B cell homeostasis under basal conditions. In IL-38-deficient (IL-38 KO) mice, we did not observe differences in global B cell levels compared to in WT mice in lymphoid organs such as bone marrow (BM), blood, spleen, and inguinal lymph nodes (LNs) (Figure 1A–D and Figure S1). Nevertheless, we observed a general increase in plasma cells in IL-38 KO mice (Figure 1E and Figure S1). Increased plasma cell levels in IL-38 KO mice may suggest a function of IL-38 in the regulation of antibody production. We, therefore, evaluated the concentrations of different immunoglobulin (Ig) isotypes in the blood of WT and IL-38 KO mice. However, despite higher levels of plasma cells, IL-38 KO mice showed lower antibody concentrations, with a reduction in IgA levels reaching statistical significance and IgM levels showing a clear tendency of reduction (Figure 1F). These data are consistent with the view that IL-38 might affect either plasma cell differentiation or antibody production. We next aimed at addressing this question in a human setting.

### 2.2. IL-38 Is Expressed in Tonsillar B Cells and Epithelium

**Figure 2:**
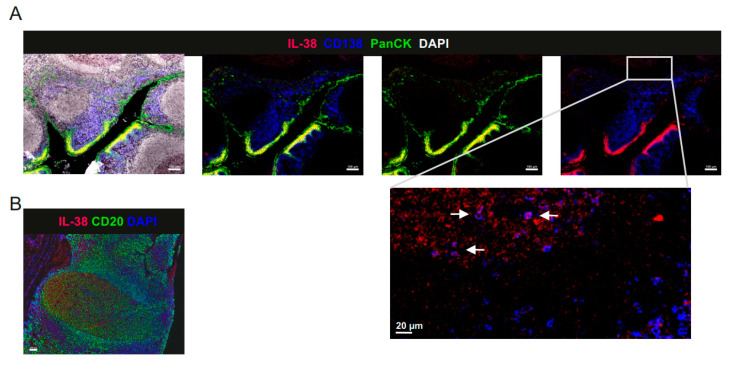
IL-38 expression in human tonsils. (**A**) Tonsil sections were stained for CD138 (blue), Pan-Cytokeratin (PanCK, green), and IL-38 (red). Nuclei were counter-stained with DAPI. The magnification shows CD38 expression in CD138^+^ plasma cells. (**B**) Tonsil sections were stained for CD20 (green) and IL-38 (red). Nuclei were counter-stained with DAPI. Representative images of 4 donors are shown. Scale bars: 100 µm (20 µm in the magnified section of (**A**)).

Lin et al. described in their initial study that IL-38 is expressed in proliferating B cells in tonsils [1]. To further delineate which B cell subsets express IL-38 in tonsils, we co-stained IL-38 with CD20 and CD138 in human tonsils. We observed that IL-38 in tonsils, as in most tissues, was highly expressed in the epithelium (Figure 2A). However, IL38 was also present in CD20^+^ B cells of the germinal centers (GC) and in CD138^+^ plasma cells (Figure 2A,B). These data confirm B cells as a relevant source of IL-38 in human tonsils and suggest the putative role of IL-38 in B cell differentiation in GC.

### 2.3. B Cell Activation Is Not Affected by Exogenous IL-38

**Figure 3:**
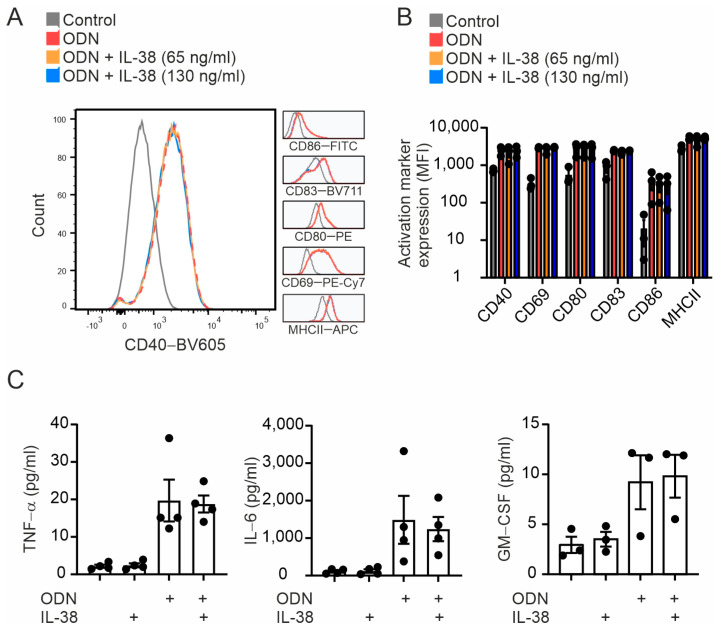
Impact of IL-38 on B cell activation. Isolated human B cells were left unstimulated (control) or activated with the TLR 9 agonist ODN 2006 (ODN), with or without an addition of exogenous IL-38 for 24 h. (**A**,**B**) Activation markers CD40, CD69, CD80, CD83, CD86, and MHCII expression levels were measured using flow cytometry. (**C**) Supernatant concentrations of TNF-α, IL-6, and GM-CSF were measured using cytometric bead arrays. Each data point corresponds to one individual donor. Data are means ± SEM.

Given the presence of IL-38 in several B cell subsets, especially in GC, where mature B cell activation, proliferation, and differentiation take place, we first explored if IL-38 influences B cell activation. To answer this, we stimulated naive CD20^+^ B cells with the TLR9 agonist CpG oligonucleotide ODN 2006 for 24 h, with or without an addition of human recombinant IL-38. We then measured the expression levels of CD40, CD69, CD80, CD83, CD86, and MCHII as B cell surface activation markers, as well as cytokine production. Using flow cytometry, we observed that while B cells responded adequately to ODN 2006, no differences in the expression levels of B cell activation markers were apparent when adding different concentrations of IL-38 (Figure 3A,B). The addition of IL-38 also did not affect B cell activation without ODN 2006 stimulation (Figure 3A,B). Moreover, IL-38 did not affect the production of tumor necrosis factor (TNF)-α, IL-6, or granulocyte-macrophage colony–stimulating factor (GM-CSF) (Figure 3C). IL-38, therefore, did not affect acute B cell activation.

### 2.4. Plasma Cell Differentiation from Naïve B Cells Is Not Affected by Exogenous IL-38

Next, we tested if long-term B cell differentiation was affected by IL-38. To investigate this, we adapted a previously developed assay to differentiate naïve B cells into antibody-producing plasma cells [28]. Different B cell subsets were identified by flow cytometry at day 4, day 7, and day 10, with relevant levels of plasma cells emerging around day 10 (Figure S2).

**Figure 4:**
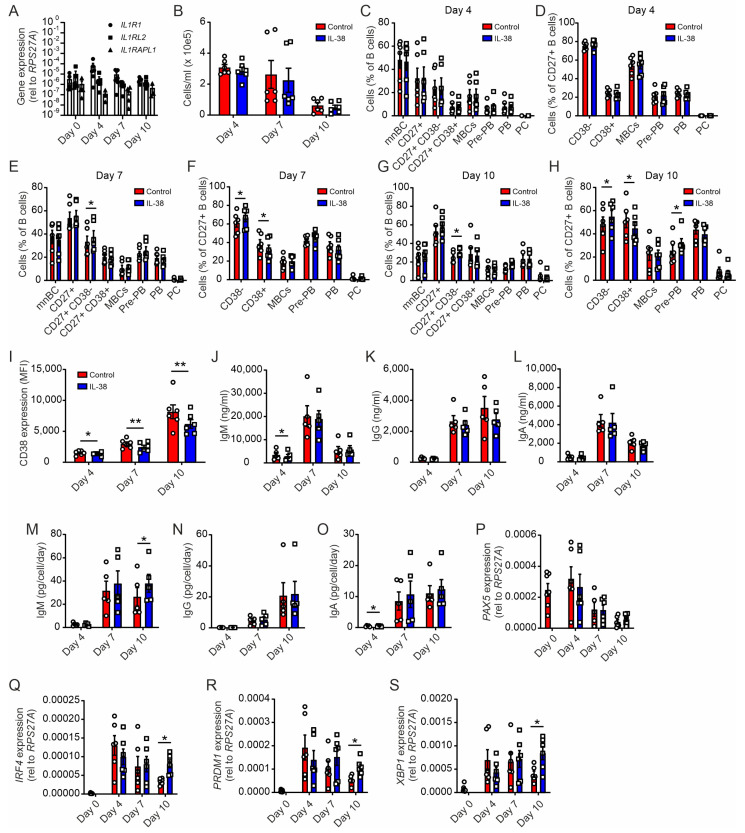
Exogenous IL-38 does not strongly affect human B cell differentiation. Isolated human B cells were differentiated for up to 10 days with or without an addition of exogenous IL-38. (**A**,**P**–**S**) RT-qPCR was used to measure mRNA expression levels of *IL1R1*, *IL1RL2*, *IL1RAPL1, PAX5, IRF4, PRDM1*, *and XBP1*. (**B**) Flow cytometry was used to measure total cells/mL at day 4, day 7, and day 10. The B cell subsets mnBC, CD27^+^, CD27^+^ CD38^−^, CD27^+^ CD38^+^, MBCs, pre-PB, PB, and PC at day 4 (**C**), day 7 (**E**), and day 10 (**G**), and CD38^−/+^, MBCs, pre-PB, PB, and PC in CD27^+^ cells at day 4 (**D**), day 7 (**F**), and day 10 (**H**). (**I**) CD38 expression levels on total B cells at days 4, 7, and 10 are shown as mean fluorescence intensity (MFI). ELISA was used to measure total IgM (**J**), IgG (**K**), and IgA (**L**) concentrations in the supernatant, which was used to calculate the respective antibody concentrations per cell/per day (**M**–**O**). Each data point corresponds to one individual donor. Data are means ± SEM. * *p* < 0.05; ** *p* < 0.01. *p*-values were calculated using multiple *t*-tests with FDR correction.

After establishing the B cell differentiation assay, we first asked if putative IL-38 receptors were expressed in B cells at various differentiation stages. We observed that the genes for all three putative IL-38 receptors, i.e., *IL1R1*, *IL1RL2*, and *IL1RAPL1*, were expressed by B cells and that their expression levels did not change significantly throughout the differentiation process (Figure 4A). Adding IL-38 during differentiation, we did not observe significant changes in total cell numbers. When looking closer at B cell subsets, which we defined as mature naïve B cells (mnBC), CD27^+^ B cells, CD27^+^ CD38^−^ B cells, CD27+ CD38^+^ B cells, memory B cells (MBCs), pre-plasmablasts (pre-PB), plasmablasts (PB), and plasma cells (PC) (Figure S2), only the CD27^+^ CD38^−^ population was significantly increased at day 7 and day 10 following IL-38 addition (Figure 4C–G). This effect seemed to reflect a rather general decrease of CD38 expression during B cell differentiation (Figure 4I). Thus, exogenous IL-38 appeared to suppress long-term activation of B cells during the differentiation process.

### 2.5. Exogenous IL-38 Enhances IgM Production

Following this observation on long-term activation and differentiation of B cells to plasma cells, we wondered if exogenous IL-38 would affect antibody production. We measured the concentrations of IgM, IgG, and IgA released into the supernatant of differentiating B cells by ELISA but did not observe any differences in total antibody production with or without an addition of IL-38, except for a slight decrease in IgM levels at day 4 (Figure 4J–L). However, when normalizing antibody production per cell and per day, we observed an enhanced IgM production in the presence of IL-38 compared to in controls (Figure 4M). This was not the case for IgG or IgA (Figure 4N,O). Increased IgM production coincided with a higher mRNA expression of markers of differentiated plasma cells—*IRF4*, *PRDM1*, and *XBP1*—following IL-38 addition, while expression of *PAX5*, which was decreased in plasma cells, was not altered (Figure 4P–S).

### 2.6. Early Silencing of IL-38 Promotes Plasma Cell Differentiation and Dampens Antibody Production

**Figure 5:**
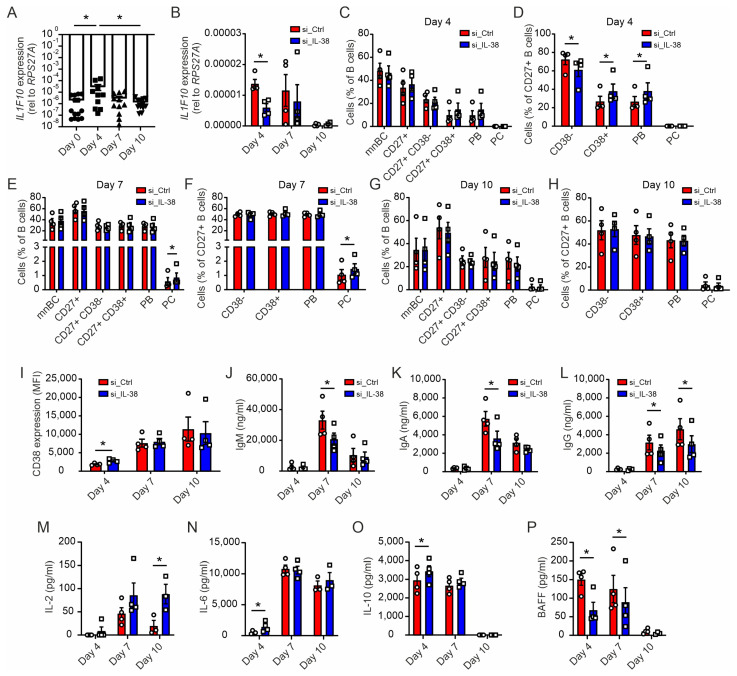
Endogenous IL-38 affects plasma cell differentiation and antibody production. Isolated human B cells were differentiated for up to 10 days with an addition of a non-targeting siRNA (si_Ctrl) or a siRNA pool targeting IL-38 (si_IL-38). (**A**,**B**) RT-qPCR was used to measure mRNA expression levels of *IL1F10* at day 0, day 4, day 7, and day 10. Flow cytometry was used to measure the B cell subsets mnBC, CD27^+^, CD27^+^ CD38^−^, CD27^+^ CD38^+^, MBCs, PB, and PC at day 4 (**C**), day 7 (**E**), and day 10 (**G**) and CD38^−/+^, MBCs, PB, and PC within CD27^+^ cells at day 4 (**D**), day 7 (**F**), and day 10 (**H**). (**I**) CD38 expression levels at days 4, 7, and 10 are shown as the mean fluorescence intensity (MFI). (**J**–**L**) ELISA was used to measure total IgG, IgM, and IgA concentrations in supernatants. (**M**–**O**) IL-2, IL-6, and IL-10 concentrations in supernatants were measured using cytometric beads arrays. (**P**) BAFF concentration in the supernatant was measured using LegendPlex. Each data point corresponds to one individual donor. Data are means ± SEM. * *p* < 0.05. *p*-values were calculated using multiple *t*-tests with FDR correction.

Overall, exogenous IL-38 only mildly affected plasma cell differentiation and did not explain differences in B cell homeostasis observed in mice (Figure 1). As previously mentioned, tonsillar proliferative B cells were described to express IL-38 [1]. Moreover, we previously obtained indications for an intracellular role of IL-38 in different cell types [3,9]. When investigating the expression of IL-38 at the mRNA level during B cell differentiation from day 0 to day 10 using RT-qPCR, we observed a significant induction of *IL1F10* mRNA encoding IL-38 at day 4, followed by a decrease at day 10 (Figure 5A). To understand if this transient upregulation was functionally relevant for B cell differentiation into plasma cells, we knocked down IL-38 at early time points during the B cell differentiation protocol. B cells were treated with *IL1F10* siRNA, compared to a scrambled control, in specific delivery media for 24 h, followed by initiating B cell differentiation. Knockdown of IL-38 (IL-38 KD) was transient but feasible, as indicated by lower *IL1F10* mRNA expression compared to in the control at day 4 (Figure 5B). Interestingly, even though a full IL-38 KD was not achieved, we observed an upregulation of CD38 expression on the surface of CD27^+^ B cells, as well as an increase of CD27^+^ PB levels at day 4 in cells with reduced IL-38 expression (Figure 5C,D). This observation was consistent with the opposite effect on CD38 expression observed when adding exogenous IL-38. Increased CD38 surface expression was followed by an increase in PCs at day 7 in the IL-38 KD cells (Figure 5E,F). The effects of IL-38 KD on cellular surface markers were transient as expected, and thus, no further impact on cell subset distribution was observed at day 10 (Figure 5G,H). Accordingly, CD38 expression was higher on total B cells at day 4 following IL-38 KD, but not at day 7 or 10, when IL-38 levels were re-established (Figure 5I). However, despite higher amounts of antibody-producing cells upon IL-38 KD, the secretion levels of IgM, IgA, and IgG were lower in these cells at day 7, and these decreases persisted until day 10 for IgG (Figure 5J–L). Cytokine production was also affected by IL-38 KD. While some cytokines were upregulated, such as IL-2 at day 10 and IL-6 and IL-10 at day 4, the survival factor B-cell activating factor (BAFF) was decreased at day 4 and day 7 (Figure 5M–P). These data suggested that IL-38 KD in B cells early during their differentiation to plasma cells fairly well reproduced our observations in IL-38 KO mice with higher plasma cell levels but reduced antibody production capacity. The altered expression of BAFF may suggest a homeostatic response to the lack of IL-38 in B cells.

### 2.7. Absence of IL-38 Dysregulates Autoantibody Production

**Figure 6:**
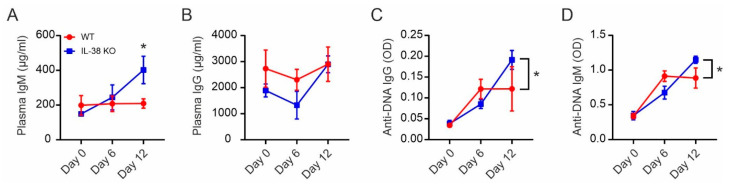
IL-38 suppresses autoantibody production in mice. WT and IL-38 KO mice received daily injections of recombinant murine IL-18 for a maximum of 10 days and were sacrificed at days 0, 6, or 12 after initial IL-18 injection. (**A**,**B**) Plasmatic IgM and IgG total concentrations were measured by ELISA. (**C**,**D**) Plasmatic IgG and IgM anti-DNA ODs were measured by ELISA. Each data point corresponds to one animal. Data are means ± SEM of *n* = 4 animals per group on days 0 and 12, and *n* = 5 per group on day 6. * *p* < 0.05. *p*-values were calculated using multiple *t*-tests with FDR correction.

Based on the potential of IL-38 to regulate antibody production described herein and its association with autoimmunity, we decided to investigate a potential involvement of IL-38 in the generation/production of autoantibodies. Injection of IL-18 was previously shown to induce autoantibody production by triggering the innate antibody response, known to be unspecific and to involve autoreactive B cell activation [29]. Following IL-18 injection, we observed increased overall production levels of IgM and IgG in IL-38 KO mice at day 12 of treatment when compared to at day 0, while these levels remained relatively unchanged in WT mice. Furthermore, IL-38 KO mice showed higher IgM concentrations at day 12 compared to WT mice and reached WT levels of IgG (Figure 6A,B). Among those antibodies, we next investigated the presence of autoantibodies directed against DNA. Interestingly, while autoantibody production peaked at day 6 in WT mice, IL-38 KO mice continued to elevate anti-DNA autoantibody production until day 12, reaching higher levels compared to WT mice at that time point (Figure 6C,D). These data align well with the protective role of IL-38 observed in auto-immune mouse models.

## 3. Discussion

IL-38, a receptor antagonist of the IL-1 cytokine family, has been described as an anti-inflammatory and pro-resolving mediator [2,5,6], and IL-38 polymorphisms are associated with autoimmunity [17,18,19]. Recently, IL-38 has been linked to B cells in a number of studies [9,10,11,12], but its role in B cell biology remained obscure. We discovered in this study that a global deletion of IL-38 in mice did not change naïve B cell homeostasis but increased the amount of plasma cells in lymphoid organs. However, this increase in plasma cells was negatively correlated with peripheral antibody titers. This relation between plasma cells and antibody titers was unexpected and raised questions about the function of IL-38 in the generation of antibody producing cells. To explore this issue, we used a human in vitro model of primary B cell activation and differentiation, to benefit from higher amounts of material accessible from human blood.

In contrast to other (immune) cell subsets such as macrophages and T cells [2,5,6,30], exogenous IL-38 had minor effects on B cell activation and differentiation. While IL-38 did not affect acute B cell activation in response to TLR9 stimulation and CD40 ligation, addition of IL-38 appeared to regulate the long-term activation during differentiation and modulated CD38 expression at the surface of B cells. Exogenous IL-38 reduced the transition of CD27^+^ to CD27^+^ CD38^+^ B cells, which is an important step toward plasma cell differentiation. CD38 expression on activated B cells plays a major role in the efficacy and maintenance of their activation. Additionally, CD38 ligation has been shown to promote IgM secretion and B cell proliferation [31]. Therefore, our observation does not concur with the current literature, as we observed higher IgM level despite a lower expression of CD38. This might suggest that in our system, the influence of IL-38 on CD38 expression does not matter for antibody secretion from plasma cells. Indeed, even though IL-38 affected CD38 expression, it did not affect the final amount of plasma cells. Interestingly, IL-38 KD in B cells increased CD38 levels, suggesting a high sensitivity of CD38 expression to IL-38. The presence of B cells expressing higher levels of CD38 in lymphoid organs was observed in B-CLL patients compared to in healthy individuals and was considered as a marker of disease aggressiveness [32]. These observations may warrant investigating the role of IL-38 in diseases such as B-cell chronic lymphocytic leukaemia (B-CLL). Decreasing expression of CD38 under these conditions may be of interest. Moreover, the interaction between CD38 and the B cell receptor (BCR) is essential for proper BCR signaling, both in normal and transformed B cells [33]. Thus, endogenous and exogenous IL-38 may affect antigen recognition by B cells, which needs to be tested in future studies.

Given the discrepancy between murine data and data derived from stimulating B cells with exogenous IL-38, we investigated the intrinsic role of IL-38 in B cell differentiation towards plasma cells by transient KD. IL-38 mRNA expression was upregulated in B cells early during differentiation, and while the KD was mostly efficient in this first phase of differentiation, we observed its impact on the later functionality of plasma cells (i.e., antibody production), suggesting the potential role of IL-38 in the early priming of plasma cell function. In agreement with the data observed in mice, we observed that, despite higher numbers of PC, antibody production was negatively impacted by the early reduction in IL-38. Given the discrepancy between endogenous and exogenous effects of IL-38, with the exception of CD38 expression, one may deduce that endogenous IL-38 does not exert its function via cell surface receptors. Other members of the IL-1 family have already been described to have intracellular function on immune cells, such as IL-1α, IL-33, and IL-37 [34,35,36]. IL-38 was found to accumulate intracellularly not only in several cell types, including B cells and tonsil epithelia, but also in keratinocytes and macrophages [3,9,37]. It remains to be determined how intracellular IL-38 affects B cell differentiation. We previously observed that IL-38 promotes glycolysis in macrophages [9]. Whether an effect of IL-38 on metabolism plays a role during B cell differentiation might be of interest, since GC B cells also rely on glycolysis in vivo [38]. However, our data so far do not exclude the possibility that endogenous IL-38 acts through B cell surface receptors. The primary mode in which IL-38 signaling through its putative cell surface receptors affects cell biology suggested so far in the literature is affecting cytokine production [7,39,40]. Transient knockdown of IL-38 increased the levels of IL-2, IL-6, and IL-10 at different time points, all of which are known to promote B cell differentiation and/or survival [41,42,43]. In contrast, IL-38 knockdown decreased the levels of BAFF, which is a prominent B cell survival factor, and high levels of BAFF are associated with autoimmunity in humans [44]. These alterations in cytokine expression linked to the observed B cell phenotype after IL-38 knockdown remain to be determined.

Nevertheless, both extrinsic and intrinsic IL-38 affected the production of antibodies, most particularly the production of IgM, even though the impact of endogenous IL-38 appeared stronger. It would be of interest to understand which source of IL-38 is decisive for B cell activation/differentiation in vivo. IL-38 is present not only in B cells and plasma cells, but also in the tonsil epithelia. This could suggest that IL-38 produced by B cells is used intracellularly to prime their function and external IL-38 produced by epithelia or released from apoptotic B cells generated during GC selection [6,45] may play a modulatory role. To address this question, cell-type-specific IL-38 ablation in B cells and epithelial cells would need to be employed both at baseline as well as in vaccination models [46] or in the IL-18 model employed here.

In the latter model of IL-18-induced autoantibody production [29], which we used to test if the association of IL-38 with autoimmune diseases may be linked to B cells [9,19,30], we unexpectedly found that IL-38 KO mice produced higher levels of anti-DNA autoantibodies. Interestingly, we previously observed a tendency for higher levels of anti-MOG autoantibodies in the circulation of IL-38 KO mice in the EAE model [9]. Thus, our data indicate that IL-38 may exert its protective function in autoimmune settings by limiting autoantibody production. Again, more sophisticated studies using cell-type-specific ablation of IL-38 will be required to answer this question. This will also help to understand by which mechanism IL-38 restricts autoantibody production. Autoreactive B cells have two main sources. They can originate from an inefficient selection within the GC or from a persistence of unspecific B cells in the marginal zone of lymphoid organs [47]. The autoimmune B cell response in the IL-18 model required activation and expansion of marginal zone B cells. Interestingly, CD1d-restricted natural killer T cells (NKT cells) were shown to limit this self-reactive B-cell response, particularly after day 6 in that model [29]. IL-38 has been shown to act directly on innate-like T cells such as γδ T cells [2], which like NKT cells also play a role in the recognition of lipids presented via CD1d. Thus, ablation of IL-38 may affect NKT cell biology, thereby affecting autoantibody production in the IL-18 model. Further studies in the IL-18 model may clarify the relevant source of autoreactive B cells in IL-38 KO mice.

Taken together, these data so far show an unappreciated role of IL-38 in human and murine B cell biology. A deeper understanding of the interaction of endogenous IL-38 with plasma cell differentiation may yield additional insights into the regulation of antibody production, as well as the development of autoreactive B cells.

## 4. Material and Methods

### 4.1. Animals

The IL-38 knockout mouse strain used for this research project (129S5-Il1f10tm1Lex/Mmucd; identification number: 032391-UCD) was obtained from the Mutant Mouse Regional Resource Centre—an NIH funded strain repository—and was donated to the MMRRC by Genentech, Inc. (San Francisco, CA, USA). All mouse experiments were approved by and followed the guidelines of the Hessian animal care and use committee (FU/1201).

### 4.2. IL-18-Induced Autoantibody Production

The IL-18-induced autoantibody production model was performed as previously described by Enoksson and colleagues [29]. Two micrograms of recombinant IL-18 (Biolegend, San Diego, CA, USA) was injected i.p. for 6 or 10 consecutive days in 8–12-week-old WT and IL-38 KO male and female mice. Mice were sacrificed at day 6 or day 12.

### 4.3. Cells

Primary human PBMCs were isolated from buffy coats of anonymous donors obtained from the DRK-Blutspendedienst Baden-Württemberg-Hessen, Institut für Transfusionsmedizin und Immunhämatologie, Frankfurt am Main. B cells were isolated using a EasySep™ Human B Cell Isolation Kit (Stemcell, Vancouver, BC, Canada), which isolated B cells by negative selection. The purity of B cells was greater than 95%, as confirmed by flow cytometry. If not differently described, all experiments were performed in Iscove’s modified Dulbecco’s Medium (IMDM), containing 10% heat-inactivated foetal calf serum (FCS), 100 U/mL penicillin, 100 mg/mL streptomycin, and 50 µg/mL transferrin and bovine insulin at 37 °C with 5% CO_2_.

### 4.4. B Cell Activation and Differentiation

Activation: B cells were left unstimulated or stimulated with 20 µg/mL ODN 2006 (InvivoGen, Toulouse, France) for 24 h with 65 or 130 ng/mL of recombinant human IL-38 (aa 7-152). IL-38 (aa 7-152) was generated as described earlier [2,6].

Differentiation: B cells were cultured in a three-step culture system for a total of 10 days with different stimuli being added at day 0 (20 µg/mL ODN 2006, 100 ng/mL sCD40L, 40 U/mL IL-2, 100 ng/mL IL-10, and 20 ng/mL IL-15), day 4 (100 ng/mL IL-6, 40 U/mL IL-2, 100 ng/mL IL-10, and 20 ng/mL IL-15), and day 7 (100 U/mL IFN-α, 100 ng/mL IL-6, and 20 ng/mL IL-15). On days 0, 4, 7, and 10, supernatants were taken for further analysis, and cells were collected for FACS and qPCR analysis. On days 4 and 7, the cells were washed with phosphate-buffered saline (PBS) and plated in a new medium without changing the concentration of cells per well. rhIL-38 was added to cells at 130 ng/mL on days 0 and 7 when seeding the cells, as well as daily between days 3 and 6 at 65 ng/mL.

### 4.5. B Cell Transfection

Isolated B cells were incubated with 1 µM Accell SMARTpool Human IL1F10 siRNA (Dharmacon, Lafayette, CO, USA) in Accell siRNA Delivery Media (Dharmacon, Lafayette, CO, USA) containing 100 U/mL penicillin, 100 mg/mL streptomycin, and 50 µg/mL transferrin and bovine Insulin (1:2000). The cells were plated in 200 µL in 96-well U-bottom plates. After 24 h, 20 µL FCS was were added to each well. B cells were cultured for a total of 10 days as mentioned above. On days 0, 4, 7, and 10, supernatants were taken for further analysis, and cells were collected for FACS as well as for qPCR. On days 4 and 7, the cells were washed with PBS and plated in an IMDM medium. As a non-targeting control, Non-Targeting siRNA Pool #1 (Dharmacon, Lafayette, CO, USA) was used at 1 µM.

### 4.6. Flow Cytometry

For flow cytometry, B cells were pelleted by centrifugation, blocked with an FcR blocking reagent (Miltenyi Biotec, Bergish Gladbach, Germany) in 0.5% PBS-BSA, stained with the fluorochrome-conjugated antibodies (Table 1) and analyzed on an LSR II/Fortessa flow cytometer. Data were analyzed using FlowJo V10 (TreeStar, Ashland, OR, USA). All antibodies and secondary reagents were titrated to determine optimal concentrations. Comp-Beads (BD Biosciences, Franklin Lakes, NJ, USA) were used for single-color compensation to create multicolor compensation matrices. For gating, fluorescence minus one control were used. The instrument calibration was controlled daily using a cytometer setup and tracking beads (BD Biosciences, Franklin Lakes, NJ, USA).

### 4.7. Quantitative PCR

Total RNA from human B cells was extracted using pegGOLD RNAPure (Peqlab Biotechnologie, Erlangen, Germany), and 1 µg of mRNA was used for reverse transcription with a Maxima First Strand cDNA Synthesis kit (Thermo Fisher Scientific, Waltham, MA, USA). Quantitative real-time PCR reactions were performed with an SYBR Select Master Mix and a QuantStudio 5 Real-Time PCR System (Thermo Fisher Scientific, Waltham, MA, USA). Relative mRNA expression was analyzed based on the DDcycle threshold method and normalized to RPS27A as a housekeeping gene. All primers were purchased from Biomers (Ulm, Germany) (Table 2). Quantitect primer assays for the *IL1F10* mRNA encoding IL-38 were purchased from Qiagen (Hilden, Germany).

### 4.8. Cytometric Bead Array

TNF-α, IL-6, and GM-CSF concentrations in the supernatants were quantified using cytometric bead array flex sets (BD Biosciences, Franklin Lakes, NJ, USA). Samples were analyzed with an LSR Fortessa flow cytometer (BD Biosciences, Franklin Lakes, NJ, USA), and data were analyzed by BD Biosciences FCAP software (V1.0.1).

### 4.9. LegendPlex

The BAFF concentration in the supernatants was quantified using LegendPlex (BioLegend, San Diego, CA, USA). Samples were analyzed with an LSR Fortessa flow cytometer (BD Biosciences, Franklin Lakes, NJ, USA), and data were analyzed using FlowJo V10 (TreeStar, Ashland, OR, USA).

### 4.10. Histology

Four-micrometer-thick human tonsil sections from patients with recurrent acute tonsillitis (tonsils were not inflamed at the time of surgery) were deparaffinized and rehydrated. An Opal 7-Color Fluorescent IHC Kit (Perkin-Elmer/Akoya, Marlborough, MA, USA) was used according to the manufacturer’s instructions. Slides were stained with primary Abs targeting CD20 (Abcam, Cambridge, UK, ab9475), CD138 (Abcam, Cambridge, UK, ab130405), PanCK (Abcam, Cambridge, UK, ab7753), and IL-38 (eBiosciences, Waltham, MA, USA, 14-7385-82). A PhenoImager HT automated quantitative pathology imaging system (Akoya, Marlborough, MA, USA) was used for image acquisition, and images were analyzed using in-Form 2.5 Software (Akoya, Marlborough, MA, USA). The collection of tonsils was approved by the Ethics Committee of University Medical Center Göttingen (25/7/18).

### 4.11. ELISA

Plasmatic human and mouse IgG, IgM, and IgA levels in WT mice and IL-38 KO mice and B cell supernatants were evaluated using specific ELISA kits for each isotype according to the manufacturer’s protocol (Invitrogen, Waltham, MA, USA). In brief, 96-well plates were coated with anti-human IgG, IgM, or IgA or with anti-mouse IgG, IgM, or IgA. Mouse plasma or human B cell culture supernatants were diluted in 2-fold serial dilutions. Absorbance was measured at 450 nm and normalized to 560 nm using a Tecan Spark plate reader. Immunoglobulin concentrations were calculated using standard calibration curves.

Anti-dsDNA autoantibodies were determined as previously described by Hoyer and colleagues [48]. Briefly, 96-well plates were pre-coated with methyl-10 μg/mL BSA (Sigma-Aldrich, Saint Louis, MO, USA) for 2 h at 37 °C and subsequently coated with 10 μg/mL activated calf thymus DNA (Sigma-Aldrich, Saint Louis, MO, USA) overnight. Plasma samples were then added, and secondary anti-IgG or anti-IgM antibodies (Invitrogen, Waltham, MA, USA) were used to detect anti-DNA IgM and anti-DNA IgG. Absorbance was measured at 450 nm and normalized to 560 nm.

### 4.12. Statistics

Statistical significance was assessed using multiple t-tests followed by FDR correction or two-tailed unpaired t-tests using GraphPad Prism 9 (GraphPad Software, La Jolla, CA, USA; * *p* < 0.05; ** *p* < 0.01).

## Figures and Tables

**Table 1:** Flow cytometry antibodies.

Antibody	Clone	Provider	Antibody	Clone	Provider
Mouse			Human		
CD45-Vioblue	30F11	Miltenyi	CD20-BV510	2H7	BioLegend
CD11b-BV605	M170	BioLegend	IgD-FITC	IA6-2	BioLegend
CD19-APC-Fire750	6D5	BioLegend	CD40-BV605	5C3	BioLegend
CD38-BV510	90	BD	CD69-PE-Cy7	FN50	BioLegend
CD138-PE	281-2	BD	CD80-BV711	L307.4	BD
MHCII-APC	M5/114.15.2	Miltenyi	CD83-PE	HB15	BioLegend
CD3-PE-CF594	145-2C11	BD	CD86-FITC	BU63	Immunotools
			MHCII-APC	L243	BioLegend
			CD27-BV711	O323	BioLegend
			CD38-BV421	HIT2	BD
			CD138-PE-Cy7	MI15	BioLegend

**Table 2:** Human qPCR primers.

Primer	Forward (5′ → 3′)	Reverse (5′ → 3′)
*IL1R1*	ATGAAATTGATGTTCGTCCCTGT	ACCACGCAATAGTAATGTCCTG
*IL1RL2*	TCCCGAAGAGTTGTGTTTTGG	TGAGTGTGTCAGTATGGCTTGA
*IL1RAPL1*	ATGAAAGCTCCGATTCCACAC	TTTGGGCAAGGGAGTAATTTGT
*PRDM1*	AAGCAACTGGATGCGCTATGT	GGGATGGGCTTAATGGTGTAGAA
*XBP1*	CCCTCCAGAACATCTCCCCAT	ACATGACTGGGTCCAAGTTGT
*PAX5*	ACTTGCTCATCAAGGTGTCAG	TCCTCCAATTACCCCAGGCTT
*IRF4*	GCTGATCGACCAGATCGACAG	CGGTTGTAGTCCTGCTTGC
*RPS27A*	CTGGAAGATGGACGTACTTTGTC	CGACGAAGGCGACTAATTTTGC

## Data Availability

All data described in this study are available from the corresponding author upon reasonable request.

## Supplementary Materials

The following supporting information can be downloaded at: https://www.mdpi.com/article/10.3390/ijms24065676/s1.

## Abbreviations

B-CLL	B-cell chronic lymphocytic lymphoma
BCR	B cell receptor
BM	bone marrow
EAE	experimental autoimmune encephalomyelitis
GC	germinal center
IBD	inflammatory bowel diseases
IL	interleukin
IL-1R	interleukin-1 receptor
IL-1RAPL1	X-linked IL-1 receptor accessory protein-like 1
IMDM	Iscove’s modified Dulbecco’s Medium
KD	knockdown
LNs	lymph nodes
MBCs	memory B cells
mnBC	mature naïve B cells
PB	plasmablast
PC	plasma cells
Pre-PB	pre-plasmablasts
Ra	receptor antagonist
RA	rheumatoid arthritis
SLE	systemic-lupus-erythematosus
