# Supplementary material for: Cell Intrinsic IL-38 Affects B Cell Differentiation and Antibody Production
Source: Int J Mol Sci. 2023 Mar 16;24(6):5676. doi: 10.3390/ijms24065676 (PMC10053218; doi:10.3390/ijms24065676)
Supplement: Supplementary file 1 [file ijms-24-05676-s001.zip › ijms-2251022-supplementary.pdf]

Supplementary Figures and Figure Legends

Figure S1. Gating of mouse B cell subsets

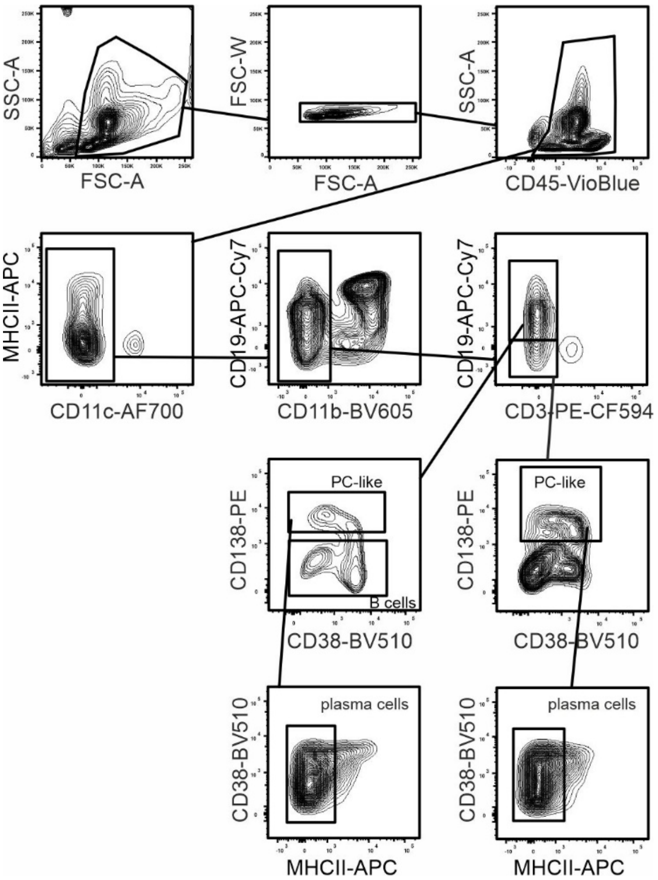

Figure S1. Gating of mouse B cell subsets. First, viable cells and cell doublets were discriminated by their SSC and FSC characteristics and further characterised as immune cells ($CD45^+$). Then, non-DCs were discriminated with CD11c and MHCII ($CD45^+$, $CD11c^-$, $MHCII^{+/-}$). Lymphoid cells were discriminated with CD11b ($CD45^+$, $CD11c^-$, $MHCII^{+/-}$, $CD11b^-$). Then, CD19hi & int and CD19low & neg were discriminated with CD19 and CD3e (CD19hi & int: $CD45^+$, $CD11c^-$, $MHCII^{+/-}$, $CD11b^-$, $CD19^{hi/int}$, $CD3e^-$; CD19low & neg: $CD45^+$, $CD11c^-$, $MHCII^{+/-}$, $CD11b^-$, $CD19^{low/-}$, $CD3e^-$). From both populations, plasma cell (PC)-like cells were discriminated with CD138 and CD38 ($CD45^+$, $CD11c^-$, $MHCII^{+/-}$, $CD11b^-$, $CD19^{hi/int}$ or $^{low/-}$, $CD3e^-$, $CD138^+$, $CD38^{int/+}$), and plasma cells were discriminated from PC-like cells with CD38 and MHCII ($CD45^+$, $CD11c^-$, $MHCII^-$, $CD11b^-$, $CD19^{hi/int}$ or $^{low/-}$, $CD3e^-$, $CD138^+$, $CD38^{int/+}$). B cells were discriminated with CD138 and CD38 from CD19hi & int ($CD45^+$, $CD11c^-$, $MHCII^{+/-}$, $CD11b^-$, $CD19^{hi/int}$, $CD3e^-$, $CD138^-$, $CD38^{+/-}$).

Figure S2

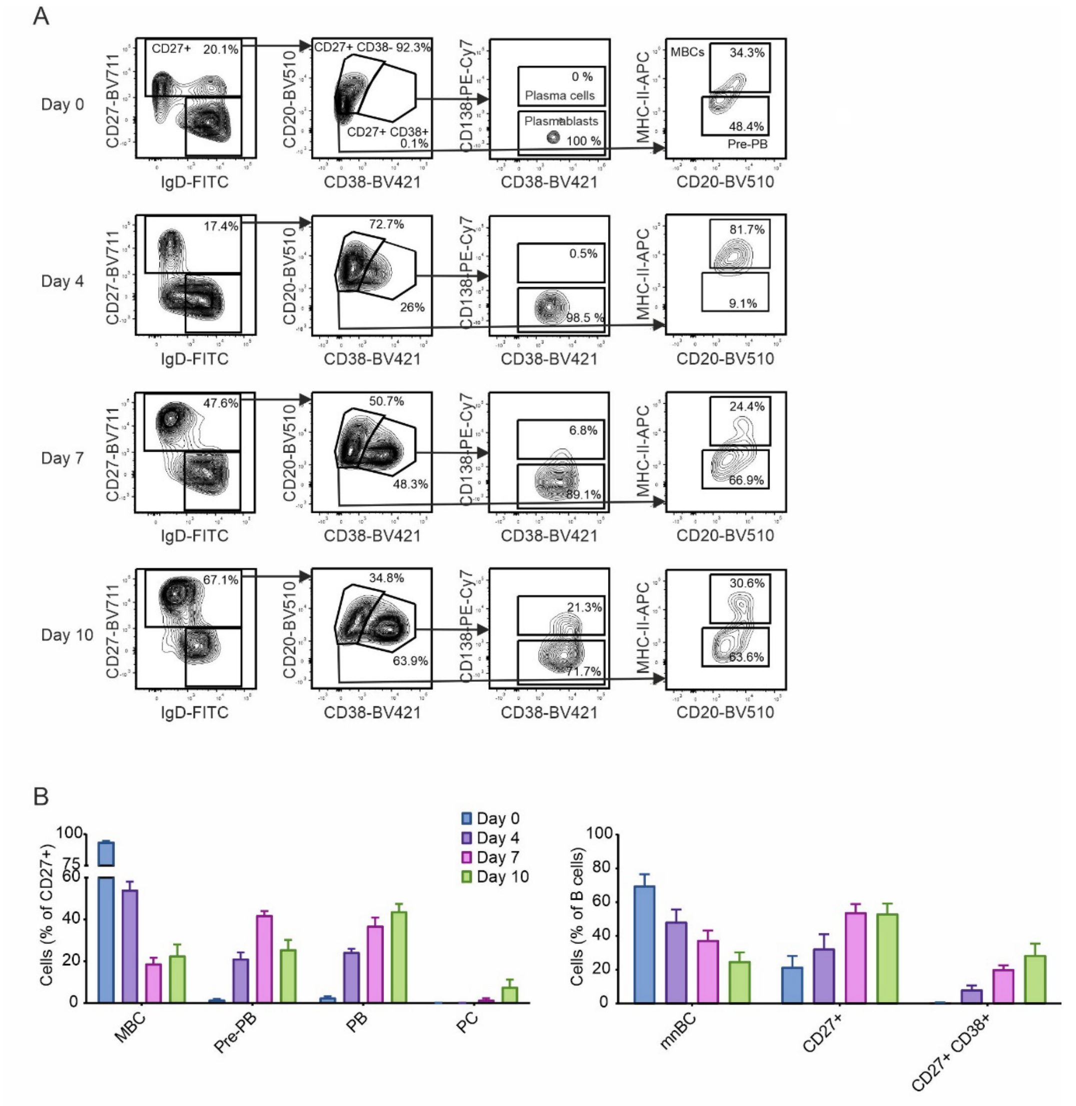

Figure S2. Human B cell differentiation to plasma cells. (A) Gating strategy for analysis of B cell differentiation to plasma cells from day 0 to day 10. First, viable cells and cell doublets were discriminated by their SSC and FSC characteristics and further characterised as B cells after removal of dead cells and non-immune cells, then discriminated with CD27 and IgD. The CD27$^+$ cell population was separated with CD20 and CD38. CD27$^+$ CD38$^+$ cells were further discriminated with CD138 and CD38. CD27$^+$ CD38- cells were discriminated with CD20 and MHCII. Subsets were then identified as such: mnBC (CD27$^-$, IgD$^+$), CD27$^+$ (CD27$^+$, IgD$^{+/-}$), CD27$^+$CD38$^+$ (CD27$^+$, IgD$^{+/-}$, CD20$^+$, CD38$^+$), MBC (CD27$^+$, IgD$^{+/-}$, CD20$^+$, CD38$^-$, MHCII$^+$), pre-PB (CD27$^+$, IgD$^{+/-}$, CD20$^+$, CD38-, MHCII$^-$), PB (CD27$^+$, IgD$^{+/-}$, CD20$^+$, CD38$^+$, CD138$^-$), PC (CD27$^+$, IgD$^{+/-}$, CD20$^+$, CD38$^+$, CD138$^+$). The shown experiment is representative for

six individual experiments. (B) Quantification of B cell subsets is shown (n = 6). Data are means ±SEM.
